# Signal peptide substitution improves immunoreactivity of *Borrelia burgdorferi* protein on *E. coli*-derived vesicles

**DOI:** 10.1007/s00253-026-13937-4

**Published:** 2026-08-06

**Authors:** David Krimer, Benjamin Taubner, Julia Squissato, Adam Hecker, Hannah Lee, Nathaniel C. Cady, Stephanie M. Curley

**Affiliations:** 1 Electrical, Computer, and Biomedical Engineering Department, Union College, 807 Union Street, Schenectady, NY 12308 USA; 2 Department of Nanoscale Science and Engineering, College of Nanotechnology, Science, and Engineering, University at Albany, 257 Fuller Road, Albany, NY 12203 USA

**Keywords:** Lyme disease, Vaccine, Outer membrane vesicles (OMVs), Lipoproteins

## Abstract

**Abstract:**

Bacterial infections account for millions of deaths every year, with many lacking preventative options. Bacterial lipoproteins are strong targets for vaccines, but are often expressed in a truncated form that may not properly recapitulate native epitopes, resulting in a reduced, or even ineffective, immune response. *Borrelia burgdorferi*, the causative agent of Lyme disease, contains numerous surface expressed lipoproteins, including outer surface protein C (OspC). OspC has been explored as a vaccine target in its recombinant form, but has resulted in a wide variation in immune response. This may be due to structural dissimilarity between the recombinant protein and the native protein expressed on the bacterium, as OspC is hypothesized to present itself in the form of a dimer containing non-linear epitopes. Presentation of OspC in a lipid membrane, such as on an outer membrane vesicle (OMV), may improve quaternary epitope presentation across dimers and better mimic the native protein. Here, we explored expression of two proteins on *E. coli*-derived OMVs: OspC derived from *B. burgdorferi* sensu lato, and a fusion protein replacing the OspC leader sequence with Braun’s lipoprotein leader sequence (Lpp). Analysis shows that replacing the OspC leader sequence with Lpp improves surface expression and immunoreactivity of the OspC protein on *E. coli*-derived OMVs exposed to recombinant anti-OspC antibodies derived from B5 and B11, as well as to *B. burgdorferi*-positive human sera. This strong antibody reactivity indicates that the OMV structure may aid in recapitulating the quaternary epitopes of OspC and provide a strategy for future development of Lyme disease vaccines.

**Key points:**

* • E. coli-derived OMVs expressed OspC when the native leader sequence was replaced with Lpp*

* • Lpp-OspC on OMV surfaces has conserved epitopes that react to B5- and B11-derived antibodies*

* • Lyme-positive serum recognizes Lpp-OspC OMVs with minimal cross-reactivity*

**Supplementary Information:**

The online version contains supplementary material available at https://doi.org/10.1007/s00253-026-13937-4.

## Introduction

Infectious diseases account for millions of deaths annually, with bacterial infections responsible for nearly one eighth of deaths worldwide. As cellular organisms, bacteria possess an inherently complex repertoire of molecular machinery that enables them to colonize a human host, evade the immune system, and resist antibiotics (Denzer et al. 2020). Bacterial lipoproteins, in particular, significantly contribute to bacterial pathogenicity by functioning as adhesins (Paulsson and Riesbeck 2018), maintaining membrane stability (Mathelié-Guinlet et al. 2020), preventing entry of antibiotics (Langevin et al. 2020), and modulating the inflammatory response (Liu et al. 2022).

Bacterial lipoproteins are classified as lipid-anchored proteins containing a modified N-terminal cysteine residue (Konovalova and Silhavy 2015). Lipoproteins are normally processed by the highly conserved localization of lipoproteins (LOL) pathway, which consists of a series of proteins containing homologues found among most Gram-negative bacteria. This signaling pathway begins with the synthesis of unprocessed lipoproteins, which contain an N-terminal LOL leader sequence composed of charged and nonpolar regions followed by a lipobox ending with a conserved cysteine residue (Babu et al. 2006; Wiktor et al. 2017). This sequence is integral for recognition of the unprocessed lipoproteins by the LOL machinery and thus processing and appropriate localization of the final lipoprotein product. In *E. coli*, the LOL pathway targets lipoproteins to the inner leaflet of the outer membrane (OM), where it is inserted by the protein LolB, demonstrating the generally internalized nature of such proteins (Tsukahara et al. 2009).

Lipoproteins located on the outer surface of bacteria significantly contribute to the cells’ ability to colonize a host and evade the immune response (Okuda and Tokuda 2011; Wilson and Bernstein 2016). In particular, *Borrelia burgdorferi* sensu latu (*Bb*), a causative agent of Lyme disease, utilizes a variety of surface-exposed lipoproteins that improve its virulence (Dowdell et al. 2017). For example, outer surface protein C (OspC), a major outer surface lipoprotein from *Bb*, significantly contributes to the bacterium’s ability to establish infection in mammals, and has been shown to enable the cell to evade phagocytosis (Anderson and Brissette 2021; Carrasco et al. 2015). OspC is usually present in the form of a homodimer and is not expressed until an infected tick feeds on the blood meal (Castro-Padovani et al. 2023).

*Borrelia burgdorferi*’s vast repertoire of surface lipoproteins significantly contributes to the bacteria’s pathogenicity, which has contributed to Lyme disease becoming the most common vector borne disease in the USA, accounting for approximately 90,000 new reported cases annually, and nearly 500,000 cases suspected but unreported (*Centers for Disease Control and Prevention, Lyme Disease Surveillance and Data* 2025). Currently, Lyme disease is treated with a 10–14 day-long course of antibiotics, highlighting the risk of significant contribution to the rise of antibiotic-resistant bacteria. If not treated in a timely manner, infection can lead to late neurological Lyme disease, Lyme arthritis, and Lyme carditis. Most early symptoms of infections are non-specific, and while the bullseye rash at the tick bite location is the indicative symptom, approximately 20–30% of patients in the USA do not exhibit this rash (Schwartz et al. 2017). This leads to a delay in seeking treatment, which reduces the effectiveness of antibiotics (Hirsch et al. 2020). Additionally, there is no available human vaccine on the market, with the previously available human vaccine, LYMERix®, ceasing production in 2002 due to high consumer costs and unfounded public fear of adverse effects (Nigrovic and Thompson 2007). Therefore, new preventative approaches for Lyme disease are needed to prevent infection in vulnerable groups and eliminate the potential for the long-term complications of Lyme disease.

OspC, discussed earlier, is of particular interest as a vaccine candidate, as it has been shown that only *Bb* expressing OspC are capable of infecting mammals (Tilly et al. 2006). While a strong target for a recombinant vaccine, historically, expression of the native full-length OspC in *E. coli* has been associated with low yields. Generating recombinant OspC by deleting at least the first 18–19 amino acids, which are identified to be the LOL signal peptide, has often resulted in an improved expression rate while maintaining the native protein core. However, such deletions can prevent post-translational modification (PTM) of the cysteine residue on the leader sequence, leading to an inability to acquire the membrane associated form (Konovalova and Silhavy 2015; Kumru et al. 2011). Furthermore, N-terminal lipidation is recognized through toll-like receptor TLR 2 by the mammalian immune system, highlighting additional immunostimulatory properties that may be provided by the native lipidated form of OspC (Nguyen et al. 2023). Therefore, expression of the truncated OspC does not fully capture the native membrane bound and lipidated form of the protein, which may affect its immunoreactivity.

Here, we aimed to recombinantly express OspC on the surface of outer membrane vesicles (OMVs), spherical bilayered (100–300 nm) extracellular vesicles secreted by Gram-negative bacteria (Sartorio et al. 2021). The surface composition of OMVs strongly resembles that of the bacteria they are derived from, and include various OM and periplasmic proteins. OMVs are also suitable as a vaccine platform due to their ability to act as adjuvants, enabling protective mucosal and systemic bactericidal antibody responses (Weynants et al. 2007). Studies have shown that the size, shape, and charge of molecules are crucial factors in antigen uptake, processing, trafficking, and lymph node localization and directly influence the immune response (Calatayud et al. 2014; Fromen et al. 2016; Kumar et al. 2015). Due to their size and surface properties, OMVs interact with antigen presenting cells (APCs) and have been shown to induce a strong and protective immune response in animal models, even when used in combination with poorly immunogenic antigens (Watkins et al. 2017). OMVs also have a high safety profile when appropriately modified (Mamat et al. 2015).

Presentation of antigens on the surface of OMVs rather than expression in the lumen has also been shown to elicit a more robust immune response. Various methods of achieving surface localization of antigens on OMVs have been developed, including the application of autotransporters such as hemoglobin binding protease (Hbp; Daleke-Schermerhorn et al. 2014), fusion to the C-terminus of the transmembrane domain of outer protein A (OmpA) and lipoprotein (Lpp-OmpA; Weyant et al. 2023), or fusion to the N- or C- terminus of cytolysin A (ClyA; Kim et al. 2008). These display systems have had varying levels of success, often dependent on the size and complexity of the fusion partner as well as the strain of bacteria used. Furthermore, the conformation of these fusion proteins may not accurately present non-linear epitopes due to the potential disruption of the antigen tertiary and quaternary structures, which are often essential for eliciting a protective immune response (Nicchi et al. 2021). Expression of certain constructs can also be associated with low yields, further preventing application of these systems in vaccine development.

In this study, rather than utilizing transmembrane protein fusion partners, we investigated the LOL pathway as a means of delivering OspC to the surface of *E. coli*-derived OMVs. Due to distinct differences in signaling pathways between *E. coli* and *Bb*, and therefore poor expression of native OspC in *E. coli*, we substituted the OspC signal peptide with that of Braun’s lipoprotein (Lpp). Lpp is the most abundant protein in *E. coli*, with approximately 1 million copies per cell (G. W. Li et al. 2014). In *E. coli*, Lpp covalently anchors the OM to peptidoglycan, regulating the width of the periplasmic space and thus the mechanical properties of the cell (Mathelié-Guinlet et al. 2020). Here, we show that substituting the native LOL signal peptide of OspC to that of Lpp improves the expression rate and surface localization of OspC in the lipidated form on *E. coli*-derived OMVs. After verification, OspC OMVs were evaluated for their immunoreactivity with OspC-specific serum and antibodies. Our results show that Lyme-positive serum and Lyme-specific antibodies have strong reactivity against OMVs containing the Lpp-substituted OspC over the native OspC OMVs, indicating potential application as a vaccine against Lyme disease.

## Materials and methods

### Plasmid construction and production of native OspC and Lpp-OspC

Native OspC (amino acids 1–210, Q07337) from B31 *B. burgdorferi* and Lpp-OspC with amino acids 1–18 of OspC (Q07337) substituted with amino acids 1–20 from major outer membrane lipoprotein Lpp (P69776) from K12 *E. coli* were expressed using *Clearcoli* ΔnlpI (Kanamycin resistance) in pET-21a (+) plasmids (Genscript). Both proteins contained a 6xhis tag on the C terminus for detection purposes. Full sequences for each recombinant protein and the restriction sites used are provided in the Supplementary Information. Bacterial transformation was done using SOB Outgrowth medium (New England BioLabs) according to New England BioLabs transformation protocol using 50 ng of plasmid.

### Bacterial culture conditions

*Clearcoli* ΔnlpI (CC), a hypervesiculating, attenuated strain of *E. coli* obtained from David Putnam at Cornell University (Watkins et al. 2017), were grown at 37 ℃ in Luria–Bertani (LB) broth (BD Biosciences) containing 50 μg/mL kanamycin with shaking at 225 RPM. When required, ampicillin was added to a final concentration of 50 μg/mL. Transformed cultures grown overnight were sub-cultured to OD_600_ = 0.08 and when they reached mid-log phase growth (OD_600_ = 0.4–0.6), protein expression was induced with 1 mM final concentration of β-d-1-thiogalactopyranoside (IPTG) (Sigma–Aldrich), as described by Watkins et al. (2017). Empty OMVs (derived from CC containing no plasmid) were used as a control.

### OMV preparation and isolation

After induced secondary cultures were grown for ~ 18 h, cells were spun down for 15 min at 3500 RPM. The supernatant was vacuum filtered using Nalgene Rapid-Flow PES 0.2-µm filter (Thermo Fisher Scientific). To isolate OMVs, the supernatant was further centrifuged using an SW 28 swinging bucket rotor and Optima XE-90 Ultracentrifuge (Beckman Coulter) at 26,000 RPM for 3 h at 4 ℃. The particles were collected using 1 mL of PBS (pH 7.4).

### OMV characterization

Total OMV protein content was determined using a Pierce BCA Protein Assay kit according to the manufacturer’s protocol (Thermo Fisher Scientific). All analyses in this study were calculated using total OMV protein content. Particle size and count were evaluated using dynamic light scattering (DLS) (Malvern Zetasizer Ultra). Western blot and SDS-PAGE were performed to confirm the presence of the OspC proteins and assess the relative protein expression rate. OMVs mixed with 4 × Laemmli sample buffer (Bio-Rad) and 2.5% β-mercaptoethanol were boiled for 3 min, then loaded into gels containing 12% acrylamide and run at 65 V for 25 min followed by 110 V for 70 min. If used for SDS-PAGE, gels were then stained with Coomassie blue (R-250, G Biosciences) and destained overnight in 10% acetic acid. For Western blotting, proteins were transferred from the gel to a PVDF membrane (Bio-Rad) for 60 min at 110 V at 4 ℃. Blots were washed with TBS-T (Tris buffered saline-Tween 20, 0.1% w/v; pH 7.4), then blocked for 1 h in 5% milk in TBS-T at room temperature (RT). Blots were then probed for the 6 × -histidine tags on the recombinant proteins using a mouse anti-His antibody (clone HIS.H8, 1:3000, Sigma-Aldrich) overnight at 4 ℃. The following day, blots were washed, incubated with goat anti-mouse HRP secondary antibody (1:5000, Sigma-Aldrich) for 2 h at RT, washed again, and developed using Clarity Western ECL Substrate (Bio-Rad). SDS-PAGE gels and Western blots were visualized using a ChemiDoc MP Imaging System (Bio-Rad).

### Binding to OspC-specific antibodies

In addition to probing for the histidine tags, recombinant proteins were evaluated for their binding to OspC-specific B5 and B11 human monoclonal antibodies (provided by Nicholas Mantis, Wadsworth Center, New York State Department of Health, Albany, NY). The B5 antibody used in this study is a recombinant chimeric derivative of the original mouse B5 antibody (Rudolph et al. 2023), and the B11 antibody used in this study is a recombinant chimeric derivative of the original human-derived bactericidal monoclonal antibody B11 against OspC (Rudolph et al. 2024). Both antibodies appear to span the homodimer interface of OspC (Rudolph et al. 2023, 2024). As OspC may be found in the dimeric conformation on the surface of *Bb*, reactivity to these antibodies may indicate that the recombinant OspC proteins on the surface of OMVs are in this dimeric form. This binding interaction was evaluated in two different ways. First, Western blot was run as described above, with the B5 (0.4 µg/mL) or B11 (0.8 µg/mL) human IgG1 as the primary antibody and a goat anti-human HRP antibody as the secondary antibody (1:10,000). In addition to a denaturing Western blot, OspC-OMV samples were analyzed by a non-denaturing dot blot to evaluate recognition of OspC protein on intact OMVs by B5 or B11 antibodies. Briefly, samples (Lpp-OspC OMVs, Native OspC OMVs, and Empty OMVs) were diluted to various concentrations then blotted (2 µL) onto nitrocellulose membrane (Whatman). After the samples dried, the membrane was blocked in 5% milk for 30 min in TBS-T and incubated for 1 h with B5 (0.4 µg/mL) or B11 (0.8 µg/mL) antibody. Membranes were then washed in TBS-T, incubated with secondary antibody (goat anti-human HRP, 1:10,000; Millipore Sigma, AP112P) for 30 min, developed, and visualized in the ChemiDoc MP Imaging System using chemiluminescence.

### Proteinase K accessibility assay

The proteinase K accessibility assay (PAA) was performed to assess the surface localization of the protein constructs expressed on the OMVs (Irene et al. 2019). Briefly, OMVs were diluted to a concentration of 30 µg/mL (total protein content) in 20 mM Tris–HCl (pH 8.0) and incubated on ice for 15 min, followed by addition of 0.1 mg/mL proteinase K (PK; Thermo Fisher Scientific) and incubation in a 37 ℃ water bath for 30 min. PK activity was then inhibited by addition of 1 mM Phenylmethylsulfonyl fluoride (PMSF, Thermo Fisher Scientific) in isopropyl alcohol. As controls, OMVs were incubated similarly and either untreated, or solubilized with 1% SDS and vortexed prior to PK being added. Once all reactions were quenched with PMSF and incubated for 10 min on ice, the samples were then analyzed in Western blot as described above.

### Protein separation based on acylation

To evaluate protein acylation, Triton X was used to separate proteins using the clouding point (Irene et al. 2019). OMVs (100 µg) were diluted in PBS (pH 7.4), then ice cold Triton X-100 was added to a final concentration of 1%. The solution was then incubated at 4 ℃ for 1 h on an end over end rotor at the maximum speed setting (40 RPM). After incubation, the solution was then placed in a water bath at 70 ℃ for 10 min to achieve phase separation of Triton X-100. The aqueous phase was separated from the detergent phase by centrifugation at 13,000 g for 10 min at 4 ℃. Protein was then extracted from each phase using methanol/chloroform precipitation and analyzed by SDS-PAGE as described above.

### Evaluation of OMV-antibody interactions with a grating-coupled fluorescent plasmonic sensor

To further evaluate the immunological response to the samples, they were analyzed with a grating-coupled fluorescent plasmonic (GC-FP) biosensor platform (Centinela System, Ciencia, Inc.) that has been utilized for assessing antibody-antigen interactions, including for Lyme disease and SARS CoV-2 (Cady et al. 2021; Chou et al. 2020; Taubner et al. 2021, 2022). Briefly, target antigens are deposited as spots onto a GC-FP chip, consisting of a nano-structured silicon-based grating chip, coated with gold. Sample antibody, typically human IgG, binds to the antigen spots. The sample antibody is then labeled with an Alexa-647-conjugated secondary antibody. A detection system determines the amount of fluorescence from the secondary antibody bound at each spot.

**Fig. 1:**
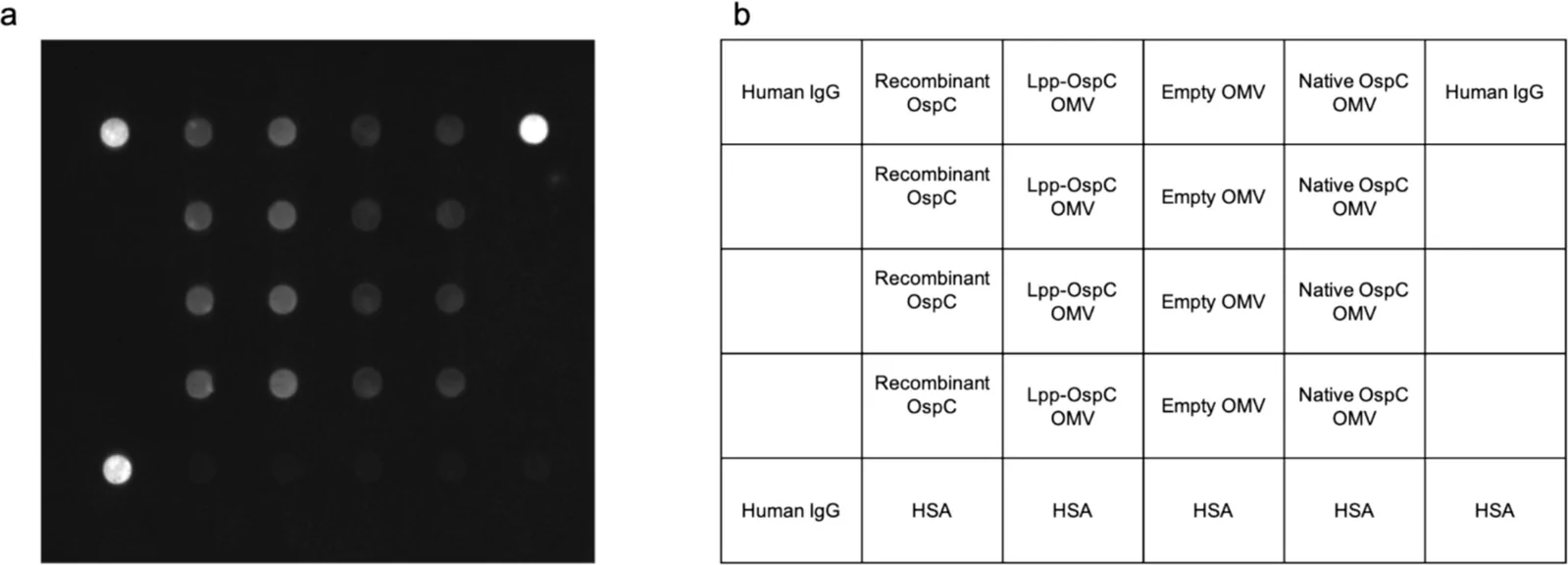
GC-FP analysis of OMVs. **a** The GC-FP biosensor chip, shown as displayed by the Ciencia Centinela instrument, contains multiple targets as described in **b**. The fluorescence intensity of each spot corresponds to the amount of antibody bound. Human IgG was used as a positive control, whereas human serum albumin (HSA) was used as a negative control for four different human samples

The OMV samples (Lpp-OspC OMVs, Native OspC OMVs, and Empty OMVs) suspended in PBS were diluted to 0.5 mg/mL in PBS. Recombinant OspC (Surmodics, Inc.), human serum albumin (HSA) (Sino Biological), and human IgG from serum (Krackeler Scientific, Inc.) were also diluted to 0.5 mg/mL in PBS. The HSA and IgG were used as negative and positive controls, respectively. All of these antigens were spotted, in volumes of approximately 1.75 nL, onto a GC-FP biosensor chip, using a SciFLEXARRAYER S3 (Scienion) (Cady et al. 2021; Chou et al. 2019; Taubner et al. 2021, 2024). The spotting pattern (Fig. 1) was designed to ensure minimal cross-reactivity, given a flow from bottom to top.

GC-FP biosensor chips were assembled with an adhesive gasket that defined the flow chamber and a polycarbonate window. Four samples were tested with the platform: Lyme-positive serological control, Lyme-positive plasma, Babesiosis-positive plasma, and Lyme-negative serological control (all purchased from SeraCare). These were diluted 1:100 in phosphate buffered saline with 0.05% Tween-20 (PBS-T; Sigma-Aldrich) immediately prior to testing. Rabbit anti-human IgG antibody labeled with Alexa-647 (Thermo Fisher Scientific) diluted 1:400 in PBS-T was used to detect human antibodies bound to the printed antigens.

A modular microfluidic system (Elveflow) was used to perform flow experiments. The flow series was as follows: PBS-T wash of 500 µL at 100 µL/min, 400 µL sample at 50 µL/min, PBS-T wash of 500 µL at 100 µL/min, 500 µL labeling antibody at 100 µL/min, PBS-T wash of 500 µL at 100 µL/min. The chip was then imaged with a Ciencia Centinela imaging system that measures defined spot fluorescence intensity in arbitrary units. Intensity values for each spot type (antigen) were averaged across all four replicates and then compared using the diagnostic algorithm described previously (Cady et al. 2021). This algorithm allows for normalization of spot intensity, where a higher ratio indicates higher degree of antibody (IgG) binding to the target spot, and thus, higher immunoreactivity of the sample toward that target antigen.

### Statistical analysis

Particle sizes from DLS and GC-FP data were analyzed using ordinary one-way ANOVA followed by Tukey’s post hoc test. All statistical analyses were conducted using Graphpad Prism10.

## Results

### Characterization of native and Lpp-OspC OMVs

**Fig. 2:**
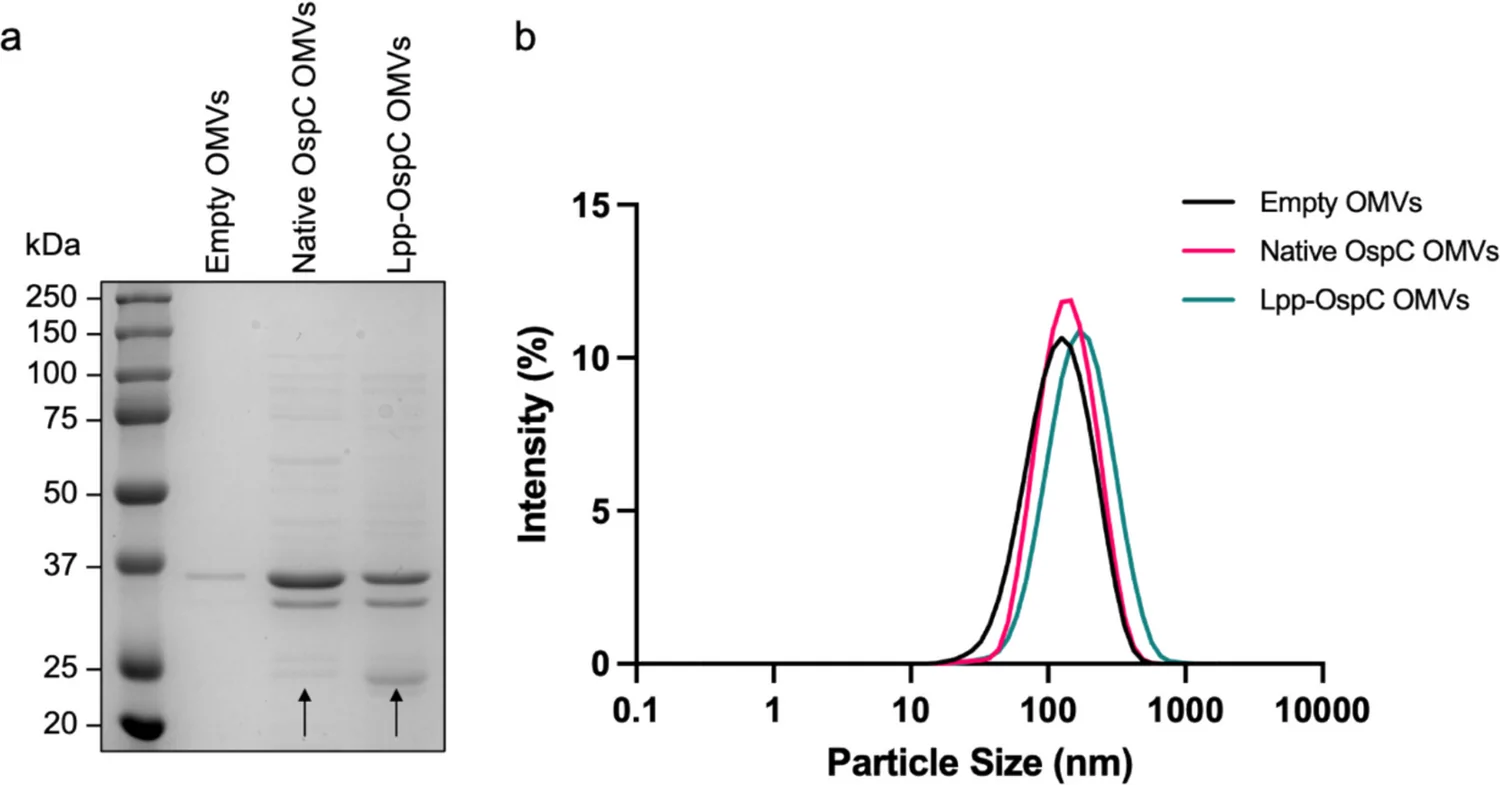
OMV characterization and analysis. **a** Expression of recombinant OspC antigens on OMVs was analyzed by SDS-PAGE. Briefly, 7.5 µg of each type of OMV (total protein content) was loaded into a polyacrylamide gel. The gel was then run at 65 V for 25 min followed by 110 V for 70 min. Gels were then stained with Coomassie blue and destained overnight in 10% acetic acid. OspC is expected around ~ 22 kDa. Substituting the native signal peptide in OspC to that of Lpp resulted in a significant increase in OspC expression. **b** OMV particle size analysis. The hydrodynamic diameter of each OMV type was analyzed using DLS to determine particle size. The reported sizes of the OMVs are shown in Table 1 (*n* = 3 separate batches with 5 runs for each sample)

It has been previously shown that fusion of the Braun’s lipoprotein leader sequence to non-lipidated proteins enables transport and insertion of the protein to the outer membrane (Irene et al. 2019). Here, we generated two different recombinant OspC proteins and evaluated the ability of hypervesiculating *Clearcoli* ΔnlpI (CC) to externally express these proteins on OMVs. The recombinant proteins were designed by synthesizing the full DNA sequence for OspC, or substituting the first 18 amino acids of the OspC sequence with the Lpp leader sequence. Each gene was then cloned into pET21a vectors and transformed into the CC strain. OMVs were purified and analyzed for total protein content using the BCA assay, and then relative OspC expression levels were analyzed using SDS-PAGE. As shown in Fig. 2a, there is much greater OspC expression on OMVs with modification of the signal peptide to Lpp in comparison to native OspC OMVs. This may indicate that substitution of the OspC signal peptide with that of the Lpp signal peptide is necessary for transport of the fusion protein to the CC outer membrane and therefore association with the vesicles. In line with these results, the BCA assay also indicated a significant increase in overall protein concentration on Lpp-OspC OMVs compared to native OspC OMVs by at least twofold. This is expected, as Lpp has been shown to increase protein expression even when paired with non-lipoproteins (Irene et al. 2019; Q. Li et al. 2023). The presence of two bands around 25 kDa in the SDS-PAGE analysis, rather than one band, was, however, unexpected, as OspC (~ 22 kDa) is found in a singular monomeric form when both denatured and non-denatured (Earnhart et al. 2011). These bands were therefore explored further in protein localization studies.

Dynamic light scattering was used to determine if particle size was impacted by expression of the recombinant proteins. Results showed that the average hydrodynamic diameters of both OspC OMVs are larger than that of empty OMVs, indicating that the recombinant proteins expressed may be surface localized (Fig. 2b, Table 1). However, only Lpp-OspC OMVs are significantly larger than both native (*p* < 0.01) and empty (*p* < 0.001) OMVs. Interestingly, the Lpp-OspC OMVs trended toward less monodispersity than the native OspC OMVs, likely due to the higher recombinant protein expression levels on the Lpp-OspC particles (Fig. 2b, Table 1).

**Table 1:** Hydrodynamic diameter of recombinant OspC OMVs measured by DLS

OMV type	Hydrodynamic diameter (nm)	Polydispersity Index (PDI)
Empty OMVs	137.0 ± 11.7	0.232 ± 0.011
Native OspC OMVs	151.2 ± 0.6	0.208 ± 0.022
Lpp-OspC OMVs	192.7 ± 11.8	0.257 ± 0.031

Standard deviation is represented by the standard error of the mean (*n* = 3 separate batches with 5 runs for each sample)

### Lyme-positive serum antibodies recognize Lpp-OspC protein on OMVs

To assess the potential serological response to Lpp-OspC OMVs, a GC-FP biosensor assay was utilized, which has been well established for quantifying the binding of human antibodies to antigens for multiple diseases. First, human plasma samples from Lyme-positive patients and patients with another tickborne disease (Babesiosis), along with Lyme-positive and Lyme-negative serological controls were assayed for antibody binding using GC-FP chips printed with a selection of diagnostically relevant *B. burgdorferi* proteins, as established in previous studies. This included purchased recombinant OspC from Surmodics. The detection ratio, discussed above, is used to compare signal from target antigens to background. A ratio of 1 indicates that the signal is comparable to the average of a negative control plus three standard deviations. Ratios above three are considered strong signals, while ratios below 1 are negative. Ratios between 1 and 1.5 are considered indeterminate. In these studies, the Lyme-positive control elicited a strong signal from most *B. burgdorferi* antigens, generating ratios as high as 7. OspC had a ratio of 3.054 (Fig. S1). The Lyme-negative control and babesiosis-positive plasma had elicited low signal from those same antigens, scoring consistently below 1.5. The OspC ratios for these samples were 1.227 and 0.826 respectively. Note that the negative control is designed to test Lyme disease assays in development, so indeterminate scores are expected. The Lyme-positive plasma had elicited signal from a smaller subset of *B. burgdorferi* antigens, with low signal from the Surmodics OspC (0.823). The difference in results from the Lyme-positive plasma could be due to differences in patient immunoreactivity to various *B. burgdorferi* antigens.

**Fig. 3:**
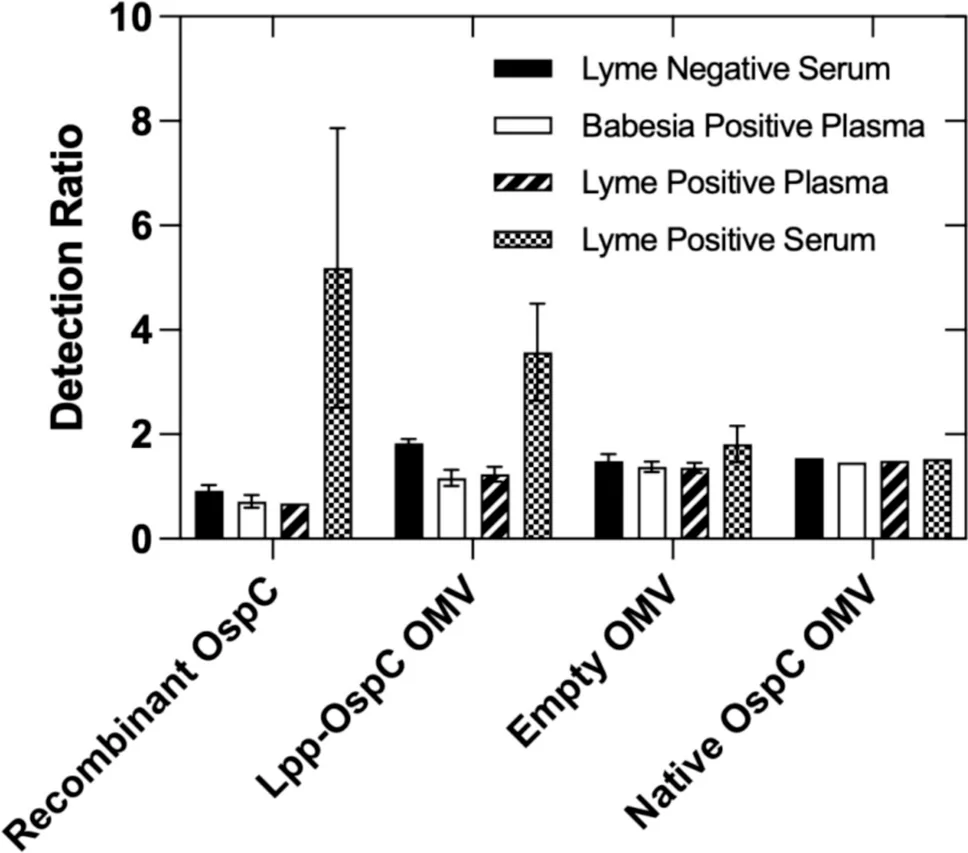
Analysis of OMV binding via GC-FP. For all samples, the Lpp-OspC OMV showed a similar profile as the Recombinant OspC, while the empty and native OspC OMVs showed negligible signal for all samples. Lyme-positive data was analyzed through one-way ANOVA with multiple comparisons, but there was no significant difference in detection ratios between groups

These samples were then tested on chips with Surmodics OspC antigen and OspC containing OMVs. Figure 3 shows the results of these tests. The high signal from the Surmodics OspC against Lyme-positive control was conserved on these chips, with an average ratio of 4.572. The Lpp-OspC OMV yielded similar strong signal for the Lyme-positive control (average ratio of 3.475), and similarly low signal for all other samples. There was, however, a large distribution in signal from the Lyme-positive control, rendering the difference statistically insignificant. The empty and native OMVs did not give noticeable signal for any of the samples tested, with the ratios to the negative control scoring similar to background, around 1.5.

### Human antibody recognition of OspC proteins on OMVs

**Fig. 4:**
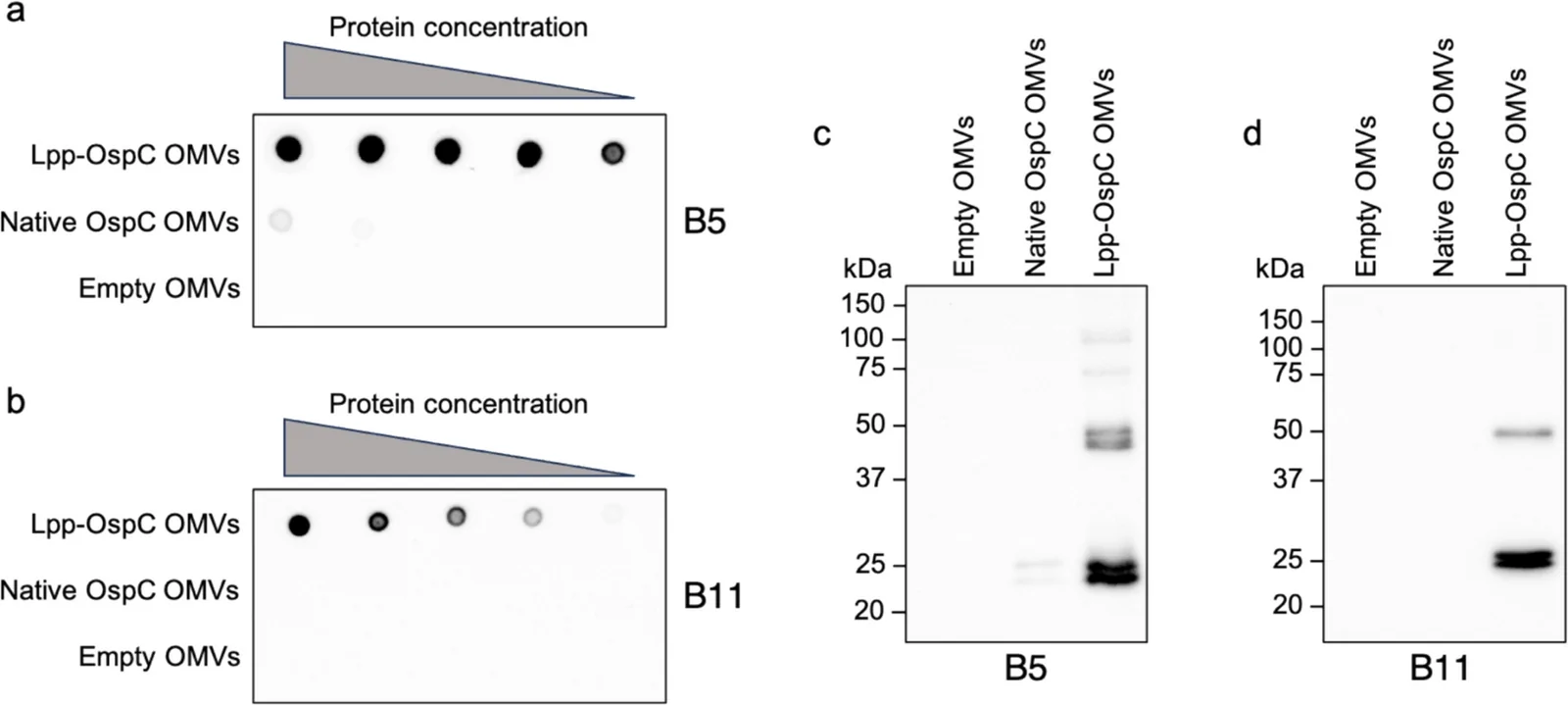
Reactivity of monoclonal human antibodies B5 and B11 with native and Lpp-OspC OMVs. **a**, **b** OMVs expressing recombinant OspC were spotted onto nitrocellulose membrane at total protein amounts ranging between 50 and 0.5 ng then probed with B5 (**a**) or B11 (**b**) IgG. Empty OMVs (OMVs containing no OspC) were similarly diluted and probed as a negative control. **c**, **d** 1.5 µg of native or Lpp-OspC OMVs was run in a denaturing Western blot and probed with B5 (**c**) or B11 (**d**) antibodies. Empty OMVs were used as a negative control

OspC natively exists in a dimeric form on the surface of *B. burgdorferi*, resulting in the formation of non-linear epitopes that span the homodimer interface. Studies indicate that these non-linear epitopes are responsible for generating highly protective antibodies, such as the OspC-specific recombinant monoclonal antibodies B5 and B11 (Rudolph et al. 2023, 2024). The IgG2a antibody B5, discovered over 20 years ago, has been shown to be protective in passive immunization studies against experimentally administered tick-transmitted infection of *B. burgdorferi* (B31) in mice (Gilmore and Piesman 2000; Mbow et al. 1999). Due to limitations in supply of the B5 antibody, Rudolph et al. (2023) generated a recombinant chimeric derivative of B5 containing human IgG_1_ Fc kappa light chains, which is the B5 antibody used in this study. B11 is a more recently discovered patient-derived monoclonal IgG that was also able to be generated recombinantly, shown to be bactericidal, and has both complement-dependent and independent activity against *B. burgdorferi* in culture (Rudolph et al. 2024). Both recombinant antibodies have been shown to complex with OspC within the monomer, as well as across the homodimer interface (Rudolph et al. 2023, 2024). In an effort to determine if the recombinant OspC proteins in this study generate the antigenic epitopes capable of binding these antibodies, particles were first assessed in a dot blot to evaluate recognition of OspC protein on intact OMVs by B5 or B11 antibodies. As can be seen in Fig. 4a and b, both antibodies react strongly with Lpp-OspC OMVs in a concentration-dependent manner. On the other hand, native OspC was undetectable by B11 (Fig. 4b) and only reacts weakly with B5 (Fig. 4a), indicating that the epitopes on these OMVs may not be in the proper conformation to be detected by these conformation-sensitive antibodies, or the protein expression levels of native OspC are too low to be detectable. Empty OMVs were also probed but, as expected, were undetectable in all cases.

To further evaluate antibody reactivity to the OMVs, a denaturing Western blot was run on Lpp-OspC, native OspC, and empty OMVs (Fig. 4c and d). Results showed that the Lpp-OspC protein directly binds to both antibodies in the monomeric form, whereas native OspC shows little binding to B5 (Fig. 4c), and no binding to B11 (Fig. 4d), indicating that the native OspC protein does not produce a readily recognizable epitope sequence when fully denatured. Alternatively, the protein content of native OspC may also be too low to be detectable here. Interestingly, there is also dimerized OspC present in the denaturing Western blot recognized by both B5 and B11 antibodies that was unable to be dissociated under normal blotting conditions. This may be caused by a hydrophobic interaction between the lipidated proteins (Earnhart et al. 2011). Taken together, these results indicate that, on OMVs, the recombinant protein Lpp-OspC may present a highly antigenic and conformationally sensitive epitope that is capable of being recognized by the immune system.

### Localization of OspC recombinant proteins on OMVs

**Fig. 5:**
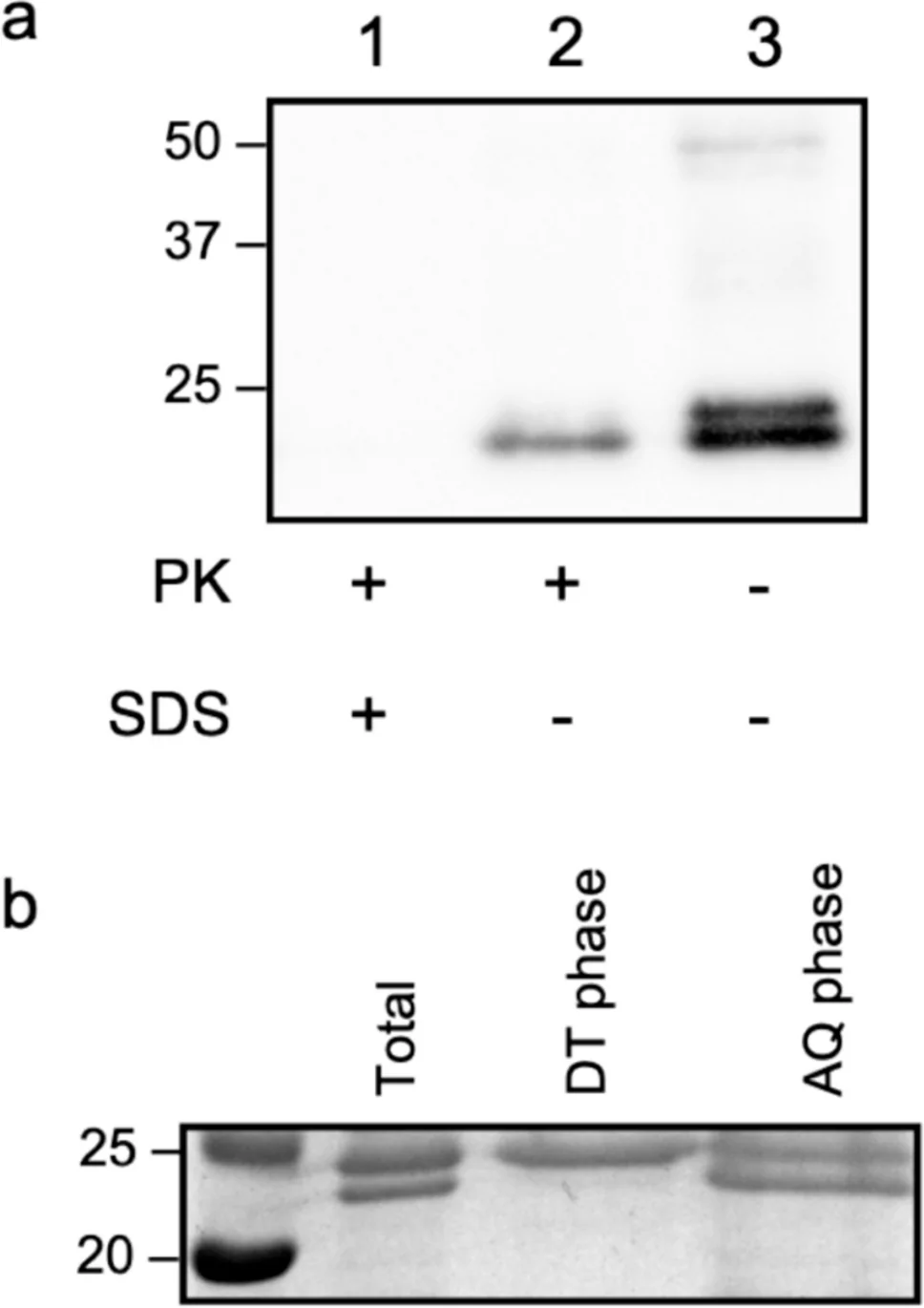
Surface localization of Lpp-OspC on OMVs. **a** Surface expression of Lpp-OspC on OMVs. The Western blot for partial proteolysis of Lpp-OspC OMVs using Proteinase K (PK) indicates surface localization of a higher molecular weight portion of Lpp-OspC on OMVs (untreated OMVs in lane 3). A total of 3 µg of proteins on OMVs was digested using PK at 37 ℃ for 30 min (lane 2). As a control, OMVs in lane 1 were preliminarily treated with SDS at a final concentration of 1% for 15 min on ice after vortexing to allow for disruption of the OMV membrane prior to PK treatment. Following PMSF treatment, samples were denatured and loaded on a 12% SDS-PAGE gel and transferred onto PVDF membrane for Western blot analysis probing for the his tag. **b** Protein lipidation analysis. Triton X-100 protein separation demonstrates that only the higher molecular weight portion of OspC is acylated (detergent phase, ‘DT’). A total of 100 µg of protein on OMVs expressing Lpp-OspC were dissolved in 1% Triton X-100 solution. After incubation at 4 ℃ for 1 h, the aqueous (‘AQ’) and detergent (‘DT’) phases were partitioned in a warm bath set to 70 ℃ and subsequently centrifuged. Hydrophobic proteins from the detergent phase and hydrophilic proteins from the aqueous phase were precipitated using chloroform/methanol extraction and separated using a 12% SDS-PAGE gel and stained with Coomassie blue

Presentation of antigens on the surface of OMVs has been shown to generate a superior immune response over interior presentation (Barat et al. 2012; Hess et al. 1996; Kang and Curtiss III 2003; Salverda et al. 2016). Therefore, the recombinant OMVs were next evaluated for surface localization of the engineered constructs using the proteinase K accessibility assay. Although OMVs containing native OspC were investigated, the Western blot did not elicit an analyzable image, likely due to consistently low yield of these OMVs and low OspC protein expression, and were therefore not investigated further. Lpp-OspC OMVs, however, indicated significant surface expression of the OspC protein. Comparable to the SDS PAGE gel, two similarly size sub-populations of OspC at approximately 25 kDa were observed upon probing with anti-histidine antibodies (lane 3, Fig. 5a). Most importantly, only the higher molecular weight band appears to be surface localized, as shown by PK induced digestion (lane 2). There also appears to be a weak band at ~ 50 kDa, which corresponds to the size of the OspC dimer and also appears to be surface localized (lane 3, Fig. 5a). Increasing the β-mercaptoethanol concentration or heat denaturation time did not resolve this dimer (data not shown).

We hypothesized that the two observed OspC bands from Lpp-OspC on the Western blots and SDS-PAGE can be attributed to partial processing of the recombinant protein by the LOL pathway. To indirectly demonstrate acylation of only one population, we used Triton X-100 to separate the proteins based on lipidation. As can be seen in SDS-PAGE in Fig. 5b, the two bands are resolved into one band with the higher molecular weight in the hydrophobic (DT) phase, which also corresponds to the surface localized population of Lpp-OspC (see Fig. 5a). The presence of two bands in the hydrophilic (AQ) phase can be attributed to incomplete extraction of acylated Lpp-OspC.

Through use of densitometry, SDS-PAGE band intensity (Fig. S2) was used to quantify the relative amount of the lipidated and non-lipidated Lpp-OspC bands compared to total protein. The results indicate that lipidated OspC accounts for approximately 15–20% of the total protein associated with OMVs, whereas the internalized non-lipidated portion accounts for about 3–5% of the total protein on the OMVs. Similarly, Irene et al. demonstrated that non-lipoprotein antigens modified to incorporate an Lpp signal peptide can be incorporated into the OM at expression rates between 10 and 20%, exceeding the expression of the same antigens without lipidation (Irene et al. 2019).

## Discussion

Outer surface protein C (OspC) is a highly prevalent lipoprotein expressed on *B. burdorferi* (*Bb*) that is required for infection in mammals (Grimm et al. 2004). OspC has often been shown to promote a robust antibody response, but antibodies against OspC vary widely in protectivity depending on the strain of *Borrelia* the sequence originates from (Marconi et al. 2020; Rudolph et al. 2023). This has been attributed to high variability in OspC sequences across a multitude of *Bb* genospecies, resulting in extensive challenges in determining a conserved epitope sequence (Baum et al. 2013; Bunikis et al. 2004; Earnhart et al. 2005; Wang et al. 1999). Furthermore, due to the quaternary structure of the protein, the most immunogenic epitopes on OspC may be non-linear or span the homodimer interface, which can be challenging to produce recombinantly.

Here, we have explored if the use of non-hazardous *E. coli*-derived outer membrane vesicles (OMVs) could provide the lipid membrane structural support to recapitulate the quaternary epitope of the OspC dimer. As cell signaling varies between species, we were also interested in determining if substituting the native OspC signal peptide with that of the signal peptide of an *E. coli* outer membrane protein, Braun’s lipoprotein (Lpp), would enable stronger expression of the recombinant protein. Indeed, this substitution led to a drastic increase in expression of the *Borrelia* antigen in CC, and although this is in line with previous studies using the Lpp signaling peptide (Irene et al. 2019; Li et al. 2023), we hypothesize that the nlpI knockout may be partially responsible for the increased protein yield in the OMVs. As nlpI knockout has been shown to decrease levels of Lpp (Banzhaf et al. 2020), the cellular machinery may be compensating for these lower protein levels by producing excess Lpp-OspC. To this end, we have identified a series of suspected alterations in the machinery of the CC that may be responsible. One study has shown that introducing this mutation affects abundance and stability of numerous proteins, particularly YidC, which is essential for embedding proteins into the inner membrane (Banzhaf et al. 2020). Upon knockout of nlpI, YidC experiences a significant drop in stability, accompanied by an increase in abundance, which may influence insertion of the OspC constructs into the membrane. Similarly, the two bands observed in SDS PAGE (Fig. 5b) and Western blot (Fig. 4c and d and Fig. 5a) around 25 kDa may also indicate that knockout of nlpI affects insertase, as well as LOL-pathway associated proteins such as LolB and SecY, which have also been known to experience a decrease in stability in ΔnlpI mutants (Banzhaf et al. 2020). Taken together, the decrease in protein stability, loss of native Lpp expression, and use of the Lpp signaling peptide in our construct may therefore all be contributing to the increased protein expression and partial processing of the Lpp-OspC protein, resulting in variable lipidated forms of the protein at around 25 kDa. However, these two subpopulations were still observed when the OspC constructs were expressed and purified from *E. coli* BL21 (DE3), indicating that knockout of nlpI is not the sole cause of the partial processing (data not shown).

In addition to improved expression, the substituted sequence also showed strong antibody reactivity in different cases (Figs. 3 and 4). Lpp-OspC reactivity to OspC-specific recombinant antibodies derived from B5 (Fig. 4a and c) and B11 (Fig. 4b and d) was much greater than that of native OspC, which only reacted with B5. Additional testing with four control samples and human serum showed that Lpp-OspC OMVs had similar reactivity to recombinant OspC in all cases. Moreover, empty OMVs and those containing a purely native sequence failed to show any reactivity. Overall, this indicates that Lpp-OspC OMVs are highly antigenic and have a strong potential for vaccine application. Furthermore, Lpp-OspC OMVs likely contain the conserved conformational epitopes recognized by the OspC-specific monoclonal antibodies B5 and B11. The B5 antibody is known to bind OspC at multiple contact points, with the complementarity-determining region spanning the homodimer interface (Rudolph et al. 2023). B11’s primary contacts are on one OspC monomer, but it also spans the homodimer interface to engage with opposing loops on a second OspC monomer (Rudolph et al. 2024). The strong reactivity with intact Lpp-OspC OMVs (Fig. 4a and b) indicates that modification with the Lpp leader sequence does not interfere with the epitope binding site, and that the OMV structure, in combination with proper signaling, may aid in recapitulating the quaternary epitope spanning the OspC-OspC homodimer. While there is some binding of each antibody to the highly denatured proteins in Western blot, this may be due to there being multiple contact points available and exposed on the Lpp-OspC proteins when denatured. However, these contact points are not all necessarily interacting in a 1:1 ratio of antibody to protein. Due to the multitude of denatured proteins present, there may be some antibody binding to different proteins’ contact points or partial binding of antibody to some of the contact points present on one protein, which would result in signal in the blot. More interestingly, due to recognition occurring despite the denaturation of OspC, this data may also support recognition of a conserved epitope sequence on the protein.

## Conclusion

Outer membrane vesicles derived from *E. coli* are valuable in vaccine development for their intrinsic properties, including safety and immunogenicity. Protein engineering further enables surface presentation of heterologous antigens on OMVs, which has been shown to elicit a protective immune response in various vaccination studies. Here, we have established application of the Lpp leader peptide to achieve increased expression along with efficient surface localization of OspC from *B. burgdorferi* on *E. coli*-derived non-pathogenic OMVs. An increase in the surface density of Lpp-OspC on OMVs, along with the possibility of presentation in its native homodimeric form, highlights the potential for the development of a protective vaccine capable of presenting complex non-linear epitopes against *B. burgdorferi* for humans. Taken together, the benefits of OMVs as a self-adjuvanting vaccine platform that mimics the bacterial surface, along with the Lpp leader peptide fusion approach for improved expression and surface localization, offer an opportunity for the development of vaccines against a wide range of bacterial pathogens.

## Supplementary Information

Below is the link to the electronic supplementary material.

Supplementary Material 1 (PDF 509 KB)

## Data Availability

All data required to evaluate the findings of this study are presented in the paper and/or the Supplementary Materials. Additional data are available from the corresponding author upon reasonable request.

## Declarations

This study contains no research involving human or animal participants.
