# Supplementary material for: Signal peptide substitution improves immunoreactivity of Borrelia burgdorferi protein on E. coli-derived vesicles
Source: Appl Microbiol Biotechnol. 2026 Aug 6;110:333. doi: 10.1007/s00253-026-13937-4 (PMC13731546; doi:10.1007/s00253-026-13937-4)
Supplement: Supplementary file 1 [file 253_2026_13937_MOESM1_ESM.pdf]

Supplementary Information for

Signal peptide substitution improves immunoreactivity of *Borrelia burgdorferi* protein on *E. coli*-derived vesicles

David Krimer, Benjamin Taubner, Julia Squissato, Adam Hecker, Hannah Lee, Nathaniel C. Cady, and Stephanie M. Curley

Stephanie M. Curley
Email: curleys@union.edu

Electrical, Computer, and Biomedical Engineering Department, Union College, 807 Union Street, Schenectady, NY 12308

This file includes:

Supplementary text
Figures S1 to S2

Supplementary Information Text

Materials and Methods

Plasmid Construction and Production of Native OspC and Lpp-OspC

The sequences shown below were cloned into the pET-21a (+) plasmid backbone using restriction sites EcoRI (GAATTC) at the 5’ end and HindIII (AAGCTT) at the 3’ end.

Lpp-OspC

5’-GAATTCTATGAAAGCTACTAAATTGGTACTGGGTGCGGTGATCCTGGGATCAACTCT
TCTTGCCGGTTGTAACAATAGTGGTAAGGACGGGAACACTTCAGCGAACAGTGCAGA
TGAGAGTGTCAAGGGGCCAAACTTAACGGAGATCTCGAAAAAGATCACAGACTCAA
ATGCAGTCCTGCTGGCAGTAAAGGAGGTAGAGGCGTTATTGTCCTCCATCGATGAAAT
TGCAGCGAAGGCGATTGGGAAGAAGATCCACCAGAATAACGGGCTGGATACAGAGA
ACAATCATAATGGGAGTTTGCTGGCGGGCGCCTATGCTATTAGTACTCTTATTAAACAG
AAGCTGGACGGTTTAAAGAATGAAGGCTTGAAAGAGAAAATCGACGCCGCGAAGAA
ATGTAGTGAAACATTCACCAACAAACTTAAAGAGAAGCACACCGATCTGGGTAAAGA
GGGAGTAACAGACGCGGATGCAAAGGAAGCTATTCTGAAAACGAATGGCACAAAAA
CCAAGGGCGCGGAGGAGCTGGGTAAGTTGTTCGAGTCCGTCGAAGTTCTGTCAAAA
GCTGCGAAGGAAATGCTGGCGAACTCCGTTAAGGAGCTTACGAGTCCGGTCGTCGCA
GAGTCCCCTAAGAAGCCCCCCGGGCACCACCATCACCATCACTAAAAGCTT-3’

Native OspC

5’-GAATTCTATGAAGAAAAACACGCTTTCTGCTATCTTAATGACTCTTTTTCTTTTTATC
TCATGTAACAACAGCGGAAAAGACGGCAACACCTCCGCGAACAGCGCCGATGAATC
CGTTAAAGGGCCGAACCTGACAGAAATCTCGAAGAAAATTACTGACTCCAACGCAGT
TTTGCTGGCGGTGAAAGAGGTCGAGGCTCTGTTGTCGAGTATTGACGAAATCGCAGC
AAAGGCGATTGGAAAGAAGATCCACCAAAACAATGGTCTTGACACCGAAAATAACC
ACAATGGTTCTCTGCTGGCGGGGGCTTATGCGATTAGTACTTTAATCAAGCAGAAATT
GGATGGATTGAAGAATGAGGGCTTGAAGGAGAAAATCGATGCTGCTAAGAAATGCAG
CGAAACGTTCACTAATAAGTTAAAGGAGAAACATACAGACCTGGGCAAGGAAGGCG
TCACAGATGCTGACGCGAAAGAGGCAATTCTTAAGACGAATGGAACCAAAACGAAG
GGGGCGGAAGAACTGGGTAAATTGTTCGAGAGTGTAGAGGTCCTTTCAAAAGCTGCC
AAGGAGATGCTTGCGAACTCTGTGAAGGAACTGACATCTCCCGTGGTTGCTGAGAGT
CCGAAAAAACCTCCCGGGCACCATCACCACCATCATTAAAAGCTT-3’

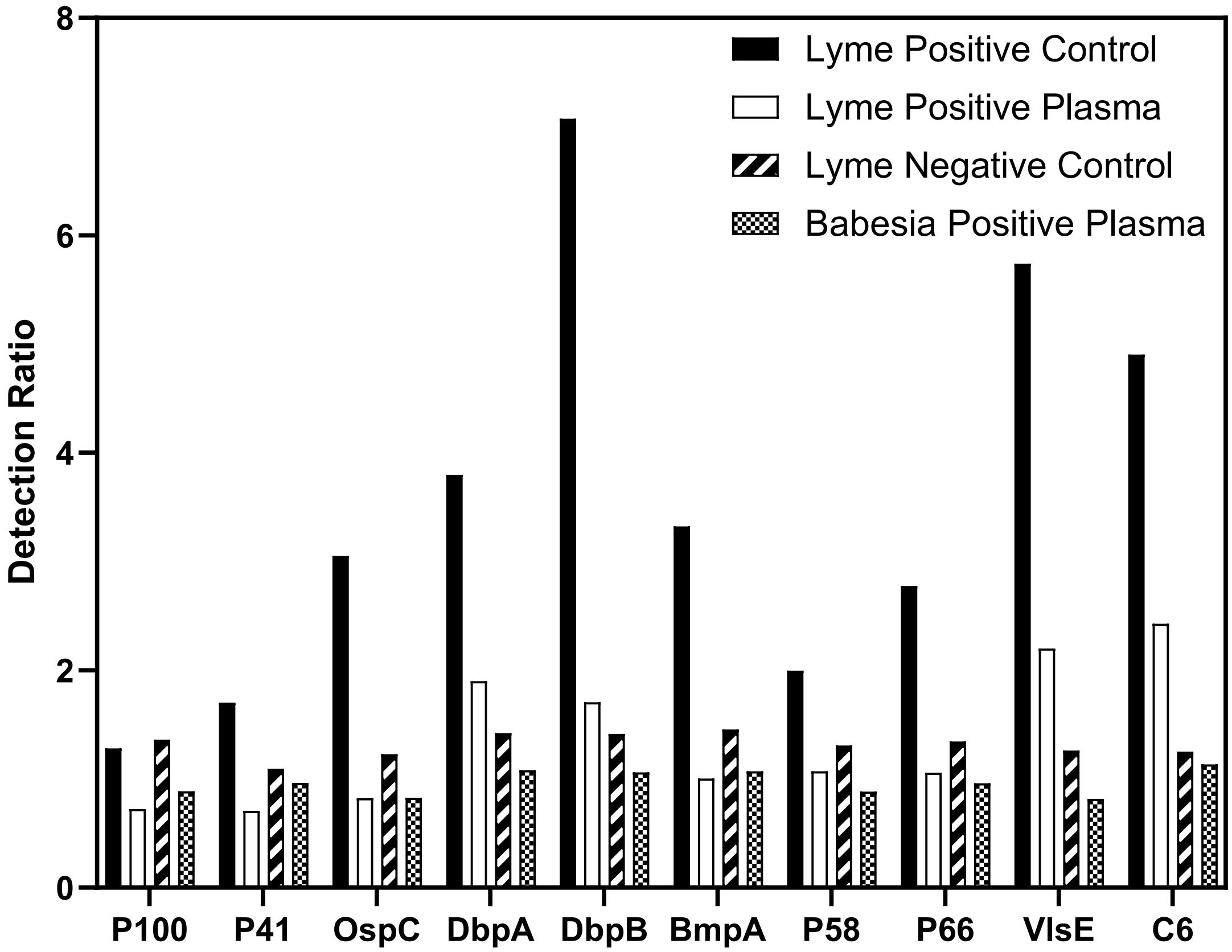

Fig. S1 Comparison of control samples with generic Lyme disease diagnostic GC-FP chip. Lyme positive serum gave strong signal for all but one antigen. Lyme positive plasma gave reduced, but measurable signal for most antigens, with weak signal from OspC. The negative control serum and babesiosis control gave no significant signal for any antigens

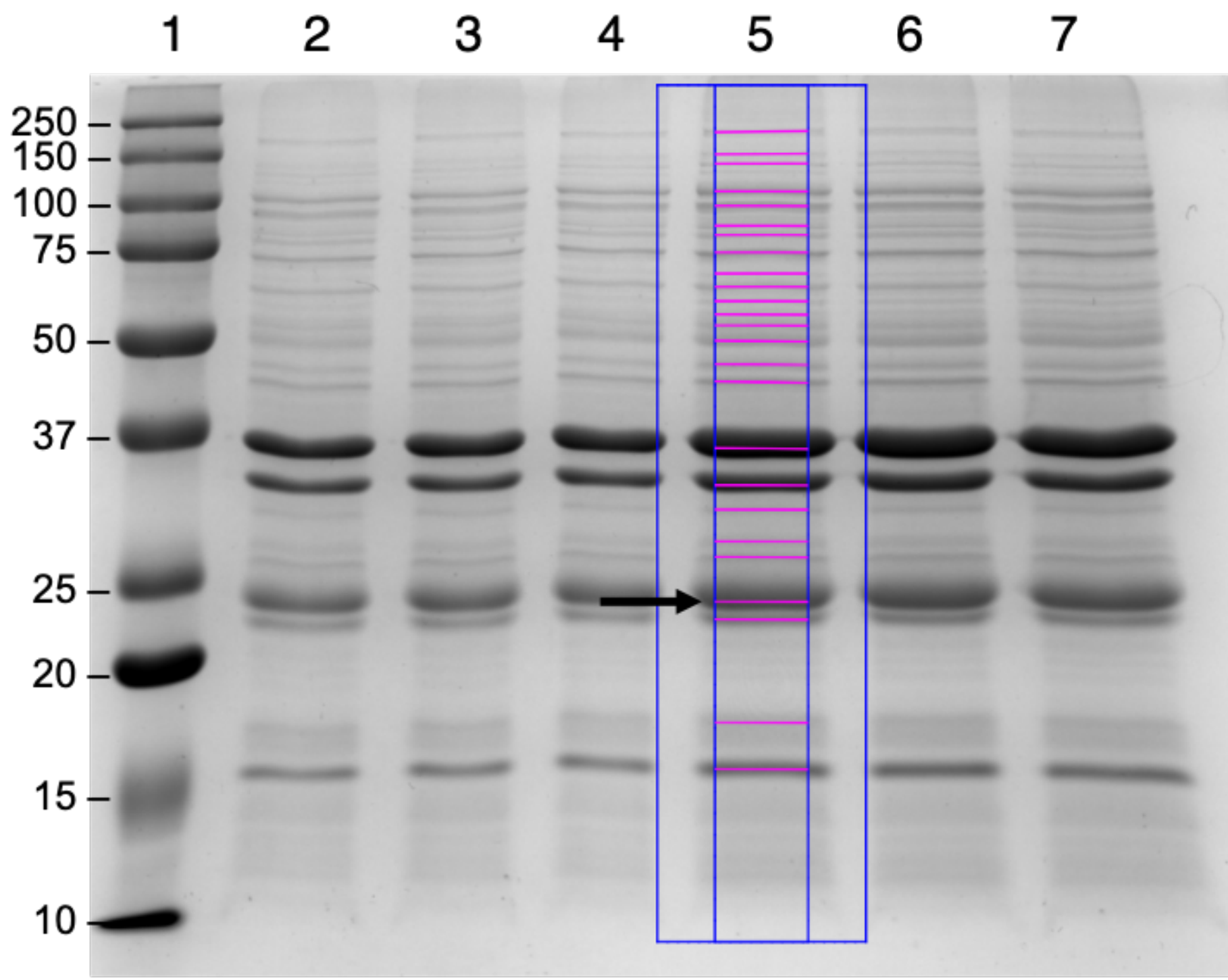

Fig. S2 Densitometry analysis of lipidated and unlipidated Lpp-OspC. Triplicates of two different amounts (5 ug in lanes 2-4 and 10 ug in lanes 5-7) of Lpp-OspC OMVs were analyzed. The molecular weight marker is shown in lane 1. Lane 5 specifically shows the bands that were analyzed for densitometry marked in pink, with the lipidated band identified by the arrow at ~25 kDa, and the unlipidated band right below the lipidated band
